# Effect of Temperature on Demographic Parameters of the Hawthorn Red Midget Moth, *Phyllonorycter corylifoliella,* on Apple

**DOI:** 10.1673/031.010.13401

**Published:** 2010-08-13

**Authors:** Abbas Amiri, Ali Asghar Talebi, Abbas Ali Zamani, Karim Kamali

**Affiliations:** ^1^ Department of Agricultural Entomology, Faculty of Agriculture, Tarbiat Modares University, P.O.Box: 14115-336, Tehran, Iran; ^2^ Department of Plant Protection, Faculty of Agriculture, Razi University, Kermanshah, Iran

**Keywords:** intrinsic rate of increase, reproduction, life table, Iran

## Abstract

The hawthorn red midget moth, *Phyllonorycter corylifoliella* (Hübner) (Lepidoptera: Gracillariidae), is one of the most serious pests of apple and pear orchards in Iran, however little is known about its biology and relationship with environmental factors. The reproduction and population growth parameters of *P. corylifoliella* were examined at six constant temperatures (15, 20, 25, 30, 33 and 35° C) on apple var. golden delicious. At 35° C, *P. corylifoliella* failed to develop beyond the first instar. The lowest (13%) and highest (64%) mortality rates of immature stages occurred at 25 and 33° C, respectively. The life expectancies (*e_x_*) decreased with increasing of age and the life expectancies of one-day-old larvae were estimated to be 38.68, 33.34, 35.11, 26.28 and 16.11 days at 15, 20, 25, 30 and 33° C, respectively. The highest intrinsic rate of natural increase (*r_m_*), net reproductive rate (*R_o_*) and finite rate of increase (λ) at 25° C were 0.100 ± 0.003, 47.66 ± 5.47 and 1.11 ± 0.00, respectively. The mean generation time (*T*) decreased with increasing temperatures from 86.86 ± 0.53 days at 15° C to 33.48 ± 0.16 days at 30° C. Doubling time (*DT*) varied significantly with temperature and the shortest doubling time was obtained at 25° C. The results of this study provide direction for future research on evaluating the performance of *P. corylifoliella* and the efficiency of its natural enemies in apple orchards under variable environmental conditions.

## Introduction

Slingerland and Crosby (1914) have estimated that about 500 insect species feed on the wood, buds, leaves, blossoms and fruits of apple trees in North America. Among the leaf-feeders are 16 species of lepidopteran leafminers in seven families (Maier 2001). Leaf-mining moths became serious pests in apple and pear orchards in the world by the end of the 1940s (Cross et al. 1999). Leafminers reduce capacity for photosynthesis and damage is expressed as premature ripening and fruit drop. Pincebourade et al. (2006) illustrate a novel mechanism by which plants might minimize losses from herbivore attacks via trade-offs between the negative impacts on photosynthesis and the positive effects of increased water use efficiency. The thermal environment of the leaf miner *Phyllonorycter blancardella* investigated in great detail by Pincebourade and Casas (2006a). They built a biophysical model to predict the temperature within a mine and suggest that this warm microclimate allows larvae to develop faster, leading to a reduced risk of attack by parasitoids. The effects of feeding activity of the leafminer *P. blancardella* on body temperature and respiration rate indicated body temperature and respiration rate increase with radiation level. Therefore, the miner is not always protected from radiations despite living within plant tissues (Pincebourade and Casas 2006b)

The family Gracillariidae is one of the largest families of plant-mining Lepidoptera with 1818 species currently recognized (De Prins and Mozaraitis 2006; De Prins 2007). *Phyllonorycter* (Lepidoptera: Gracillaridae) is one of the most species-rich genera of all Lepidoptera and has been the subject of a great deal of researche in past decades (Pottinger and Leroux 1971; Askew and Shaw 1979; Reissig et al. 1982; De Prins and Mozaraitis 2006; Lopez-Vaamonde et al. 2003, 2006). Some of them are well known as pests of fruit orchards in the Holarctic region (Baggiolini 1960; Pottinger and Leroux 1971). *P. corylifoliella* (Hübner) was first reported from Iran in 1970 and gradually spread to across north, northwest and central regions (Radjabi 1986; Modarres Aval 1997). This species is widespread in Europe and also recorded in the Near East including Asian Turkey, Georgia, Armenia, Azerbaidjan, Lebanon, Syria, Israel, Jordan, Sinai Peninsula (Egypt), the Arabian peninsula, Iran and Iraq (Olivella 2000). Most *Phyllonorycter* species are specialists, typically restricted to a single host-plant genus or, in some cases, even a single plant species. However, *P. corylifoliella* is a relative generalist species, which is known to feed on plants from six genera in two families, Betulaceae and Rosaceae. This moth, with other related species such as *P. blancardella* and *P. turanica,* has become an important pest of rosaceous trees in many apple-producing areas in Iran (Radjabi 1986). For each pest management program, an exact determination of the demographic parameters is required. Demographic parameters are important in measurement of population growth capacity of a species under specified conditions (Southwood and Henderson 2000). These parameters are also used as indices of population growth rates responding to selected conditions, and as bioclimatic indices in assessing the potential of a pest population growth in a new area (Southwood and Henderson 2000). Demographic population analysis has diverse applications for examining the dynamics of colonizing or invading species, predicting life history evolution, predicting outbreaks in pest species and estimating extinction probabilities (Granett et al. 1983; Trichilo and Leigh 1985; Carey et al. 1988; Omer et al. 1992; McPeek and Kalisz 1993; Vargas et al. 1997). Demographic information may also be useful in constructing population models and understanding interactions with other insect pests and natural enemies (Carey 1982).

The comprehensive knowledge of different biological characteristics of *P. corylifoliella* under variable environmental conditions is required for the establishment of a pest management program in apple orchards. The demographic parameters of *P. corylifoliella,* have not been studied. Therefore, the main objective of this study is to determine the relationship between various demographic parameters and temperature for *P. corylifoliella.*

## Materials and methods

### Rearing methods and experimental conditions

This study was carried out during 2007 in the Department of Entomology, College of Agriculture, Tarbiat Modares University, Tehran, Iran. Forty-two one-year-old apple trees, *Malus domestica* var. golden delicious, Borkhausen (Rosales: Rosaceae), nearly 120 cm in height were transferred to growth chambers at 25 ± 1° C, 60 ± 5% RH and 16:8 L:D. The leafminers used in the experiments were originally collected from apple orchards in the Seddeh (52°, 12′ E and 30°, 43′ N), located in the north of Fars Province of Iran. Apple leaves infested with pupae and last instar larvae were transferred into plastic containers and were kept in a growth chamber. After emergence, adults were transferred into mating cages (40×40×40 cm) containing apple seedlings for 12 h. The adult moths were supplied with fresh food (10% honey-water solution), which was sprayed on leaves during the mating period. Then, 15–20 mated female moths were released into cylindrical Plexiglas containers (40 cm diameter and 60 cm height) on apple seedlings for 12 h. The exposed seedlings were kept in a growth chamber (25 ± 1° C, 65 ± 5% RH and 16: 8 L:D) until the leaf miner population reached to desired numbers (more than 500 pairs adults). Adults obtained at 25° C were reared for one generation at each temperature (15, 20, 25, 30 and 33° C) with the same procedure before using them in the experiments.

### Survival and mortality

The effect of six constant temperatures, 15, 20, 25, 30, 33 and 35° C, on survival and mortality of *P. corylifoliella* was determined under laboratory conditions. The experiments were conducted in temperature-controlled incubators (Binder, model KBWF720, http://www.binder-world.com) operated at assigned constant temperatures. At the beginning of experiments for each temperature, 100 newly laid eggs on apple leaves were selected (1–2 eggs per leaf). Developmental stages of *P. corylifoliella* were monitored daily at 10X and survival or mortality of eggs, larvae, pupae and adults were recorded. The experiments were continued until the death of all individuals of the cohort. Based on the data of mortality and survivorship of *P. corylifoliella,* two life table parameters were calculated by the following equations (Carey 1993):


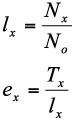


where *x* is the age in days, *l_x_* is age-specific survival rate or the fraction of individuals of the initial cohort alive at age *x, N_x_* is number alive at age *x, N_o_* is the initial number of individuals in the cohort, *e_x_* is life expectancy at age *x, T_x_* is the number of time units lived by the cohort from age *x* until the death of all cohort individuals.

### Reproduction and population growth parameters

The reproduction and population growth parameters of *P. corylifoliella* were studied at five constant temperatures including 15, 20, 25, 30 and 33° C. The leafminer moths failed to develop at temperatures of 10 and 35° C. Therefore, these temperatures were excluded from the data analysis. At the beginning of experiments for each temperature, newly emerged virgin males and females (10–20♂ × 10–20♀) were taken from those reared at different temperatures. Each couple were separately placed into detached apple leaf discs with wet cotton wool in Petri dishes (7 cm diameter, 2 cm height) and then transferred into growth chambers with a specific temperature that experiments should be conducted, relative humidity of 60 ± 5% and a photoperiod of 16: 8 L:D. Each Petri dish was observed daily and during the reproductive period, newly laid eggs were counted and then removed. This procedure continued until death of all adult females.

From the females age in days (*x*), age-specific survival (*l_x_*) and age-specific fecundity (*m_x_*), the following parameters were calculated using formula suggested by Carey (1993): gross and net fecundity and fertility rates, mean eggs per day, mean fertile eggs per day, intrinsic rate of natural increase (*r_m_*), mean generation time (*T*), finite rate of increase (λ), net reproduction rate *(R_0_)* and doubling time (*DT*).

### Data analysis

The statistical differences in various demographic parameters were tested using the Jackknife procedure (Meyer et al. 1986). This procedure is used mostly to estimate variance and bias of estimators. It is based on repeated recalculation of the required estimator, removing one sample in turn (Maia et al. 2000). It is used to quantify uncertainty associated with parameter estimates, as an alternative to analytical procedures in cases for which the last ones require very complicated mathematical derivation (Maia et al. 2000).

Algorithms for jackknife estimation of the means and variances are described only for *r_m_.* Similar procedures were used for the other parameters. The steps for the application of the method are the following (Maia et al. 2000; Zamani et al. 2006):

A) Estimation of *r_m_*, considering the survival and reproduction data for all the *n* females, referred to as true calculation. At this point, called step zero, estimates obtained are denoted as *r_m_*_(all)_ (Maia et al. 2000).

B) Repeat the procedure described in part *a* for *n* times, each time excluding a different female. In so doing, in each step *i*, data of *n*-1 females are taken to estimate parameters for each step, now named *r_m_* (*i*) (Maia et al. 2000).

C) In each step *i*, pseudo-values are calculated for each parameter, subtracting the estimate in step zero from the estimate in step *i*, For instance, the pseudo-values of *r_m_, r_m_* (*J*), was calculated for the *n* samples using the following equations (Maia et al. 2000):


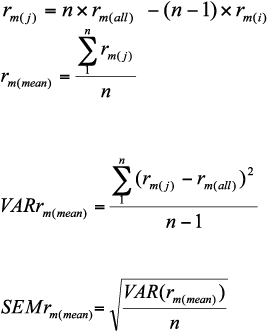


The differences in reproduction and population parameters were compared using one-way analysis of variance (ANOVA). If significant differences were detected, multiple comparisons were made using Student-Newman-Keuls (SNK) (*P*<0.05). Statistical analysis was carried out using Minitab software (MINITAB 2000).

## Results

### Survivorship, mortality and fecundity

**Figure 1.**
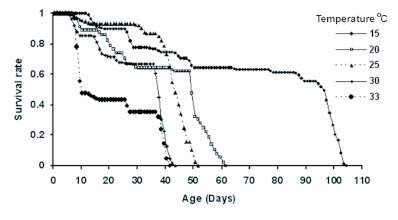
Age-specific survivorship (*l_x_*) of *Phyllonorycter corylifoliella* from egg stage to adult death at five constant temperatures. High quality figures are available online.

**Figure 2.**
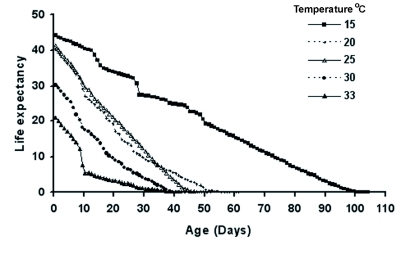
Life expectancy (e_x_) of *Phyllonorycter corylifoliella* from egg stage to adult death at five constant temperatures. High quality figures are available online.

**Figure 3.**
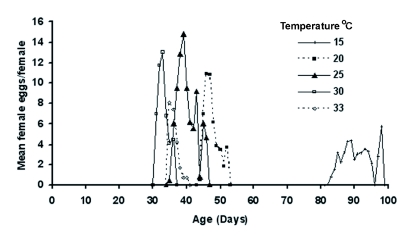
Age-specific fecundity (*m_x_*) of *Phyllonorycter corylifoliella* at five constant temperatures, (age expressed from the onset of the egg stage). High quality figures are available online.

The age-specific survivorship pattern of *P.* *corylifoliella* at different temperatures is shown in Figure 1. *P. corylifoliella* completed its development from 15 to 33° C. At 35° C, 16% of eggs reached to the first larval instar and then died. The lowest (13%) and highest (64%) mortality rates of immature stages occurred at 25 and 33° C, respectively. The immature mortality rates at 15, 20 and 30° C were 38, 36 and 33%, respectively. The highest survivorship period of *P. corylifoliella* was at 15° C (Figure 1). In general, life expectancies (*e_x_*) for *P. corylifoliella* decreased with increasing age and the life expectancies of one-day-old larvae were estimated to be 38.68, 33.34, 35.11, 26.28 and 16.11 days at 15, 20, 25, 30 and 33° C, respectively and life expectancies of adults at emergence were 7.27, 4.35, 8.49, 4.75 and 1.49 days, respectively (Figure 2). Age-specific fecundity of *P. corylifoliella* reared at various constant temperatures is shown in Figure 3. The moths reared at 15° C had a longer reproductive period than those reared at 20, 25, 30 and 33° C. There was no significant difference in the number of reproducing days at 30 and 33° C. Oviposition generally began one day after adult emergence. The mean daily reproductive rate (eggs/female/day) was 5.36 ± 0.76, 9.14 ± 2.31, 12.56 ± 2.41, 13.40 ± 3.40 and 6.80 ± 2.56 at 15, 20, 25, 30 and 33° C, respectively. Daily females' fecundities rise to a peak on days 18, 4, 6, 4 and 2 after adult emergence at 15, 20, 25, 30 and 33° C, respectively (Figure 3).

### Reproduction and population growth parameters

The effect of temperature was highly significant for the different reproductive parameters including gross (*F* = 11.41; *df* = 4; *P_value_*= 0.00) and net (*F* = 14.405; *df* = 4; *P_value_*= 0.00) fecundity rates, gross (*F* = 15.82; *df* = 4; *P_value_*= 0.00) and net (*F* = 17.51; *df* = 4; *P_value_*= 0.00) fertility rates, mean eggs per day (*F* = 10.66; *df* = 4; *P_value_*= 0.00) and mean fertile eggs per day (*F* = 13.15; *df* = 4; *P_value_*= 0.00) (Table 1). The highest and lowest values of gross and net fecundity and fertility rates of *P. corylifoliella* were found at 25 and 33° C, respectively. In general, the fecundity and fertility gross and net rates increased with increasing temperature from 15 to 25° C and then decreased at 30 and 33° C. The gross fecundity rates varied from 47.58 ± 10.68 at 33° C to 173.36 ± 16.18 at 25° C (Table 1). The maximum values of mean eggs per day and mean fertile eggs per day were estimated to be 15.74 ± 1.48 and 14.15 ± 1.32, at 30 and 25° C, respectively (Table 1).

The population growth parameters of *P. corylifoliella* at five constant temperatures are summarized in Table 2. The Net reproductive rate (*R_o_*) is the average number of female offspring produced in a lifetime by a female and was significantly different (*F* = 18.56; *df* = 4; *P_value_* = 0.00) at all temperatures according to the pattern of 25> 20> 30> 15> 33° C. The intrinsic rate of natural increase (*r_m_*) also differed significantly at various constant temperatures (*F* = 112.94; *df* = 4; *P^value^* = 0.00) and increased almost linearly with increasing temperature to reach a maximum at 25° C and then decreased at 30 and 33° C, presenting an asymmetrical domeshaped pattern. The highest *r_m_* value was calculated to be 0.100 ± 0.003 at 25° C. The temperature showed significantly effects on the finite rate of increase (λ) (*F* = 111.308; *df* = 4,; *P_value_*= 0.00), doubling time (*DT*) (*F* = 98.622; *df* = 4; *P_value_* = 0.00) and mean generation time (*T*) (*F* = 2361.503; *df* = 4; *P_value_* = 0.00) (Table 2). Like *r_m_*, the finite rate of increase was higher at 25° C than at the other temperatures. The shortest and longest values of doubling time were estimated to be 6.97 ± 0.21 and 20.57 ± 1.18 days at 25 and 15° C, respectively. Unlike the other population growth statistics, the mean generation time was the shortest at 30° C.

**Table 1.**
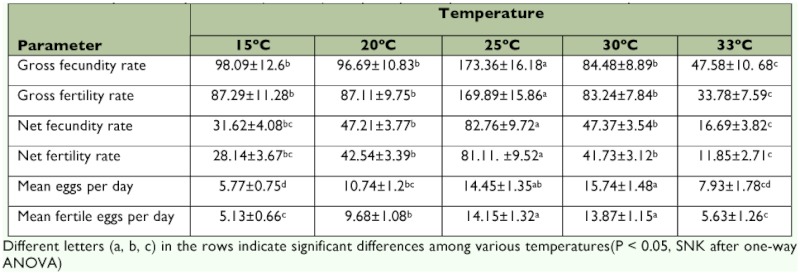
The reproductive parameters (mean±SE) of *Phyllonorycter corylifoliella* at five constant temperatures

## Discussion

Although insects do not live in a stable environment without temperature fluctuation, the results of studies under constant temperatures are still very useful in understanding the population dynamics of various insects (Summers *et al.* 1984).

This study provides realistic information on the effects of a broad range of constant temperatures on demography of *P.* *corylifoliella* on golden delicious apples, which has not been previously studied. The results revealed obvious effects of temperature on the mortality, survival and fecundity of *P. corylifoliella.* Population demographic parameters are important in measurement of population growth capacity of a species under specified conditions. These parameters are also used as indices of population growth rates responding to selected conditions and as bioclimatic indices in assessing the potential of a pest population growth in a new area (Southwood and Henderson, 2000). Temperature had a significantly influence on the various population demographic parameters. The *r_m_* is an important value, because it indicates the temperature at which the growth of a population is most favorable, and this reflects overall effects of temperature on development, reproduction, and survival (Southwood and Henderson, 2000). The greatest value of the intrinsic rate of natural increase was obtained at 25° C, indicating that this temperature is optimum for reproduction of *P. corylifoliella* and its population would proliferate very fast at 25° C. This reflected the occurrence of a high oviposition rate early in adult life at this temperature and reproduction values at this temperature were more favorable than others. Thus, 25° C may be the best choice for maintenance of a laboratory colony of *P. corylifoliella.* The lowest *R*oat 33° C resulted in heavy mortality of the immature life stages and also of adults between emergence and peak oviposition (Figure 1). The shortest value of the mean generation time was obtained at 30° C indicating that development of *P. corylifoliella* took place faster at this temperature than at the other temperatures. The findings of this study can be used for predicting *P. corylifoliella* population dynamics at different temperatures under field conditions.

**Table 2.**
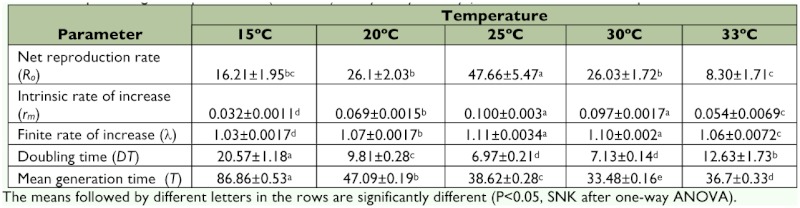
Population growth parameters (mean±SE) of *Phyllonorycter corylifoliella* at five constant temperatures.

Many models have been introduced for prediction of population dynamics of pests and their natural enemies by ecologists (e. g. Lotka-Volterra model, Nicholson-Baily model, etc), and nearly in all of these models the intrinsic rate of increase (*r_m_*) is a key parameter. Although *r_m_* has been calculated for many insects, most models are not sufficiently accurate for forecasting population dynamics. Since in many cases, *r_m_*has been calculated at a constant temperature under laboratory conditions, while in the field insects are faced with temperature fluctuations, and growth rates of insects are different at the various temperatures. In this study, the intrinsic rate of increase of the hawthorn red midget moth was calculated at various constant temperatures. Therefore, we can more accurately predict population dynamics of *P*. *corylifoliella* in the field under variable conditions.

An understanding of thermal requirements of a particular insect such as *P. corylifoliella* is important for predicting of its potential geographic distribution in different regions. Using the obtained data in this study, we will able to generate isothermal lines to predict the probable distribution regions for *P. corylifoliella* using the method of Manrique *et al.* (2008). They used the pupal lethal times (lethal time50and 90) of *Episimus utilis* Zimmerman at 0 and 5° C to develop models to predict isothermal lines with regions unfavorable for *E. utilisi* establishment.

Using a temperature-controlled environment is an essential component of mass production facilities for consistent rearing of insects for field release programs. Mass rearing of parasitoids with the leafminer as a host is being done in insectariums (Haghani *et al.*, 2006), making our findings useful for mass production of parasitoids. Likewise, survival and adult longevity measured under different temperature regimens are important for understanding leafminer invasive biology and overwintering behavior (Papadopoulos *et al.,* 1998). These factors become important when leafminers are introduced accidentally into new areas and eradication is considered (Vargas *et al.* 2000). In conclusion, this research has shown that 25° C is the most suitable temperature regime for *P. corylifoliella* population growth on apple. The results obtained during this study at several constant temperatures will be useful for future research for evaluating the performance of *P. corylifoliella* and the efficiency of its natural enemies in apple orchards under variable environmental conditions. More attention should be devoted to semi-field and field experiments to obtain more applicable results under field conditions.

## **Abbreviations**

e_x_: life expectancy
*l_x_*: age-specific survival rate
mx: age-specific fecundity
*R_o_*: net reproductive rate
*r_m_*: intrinsic rate of natural increase
*T*: generation time
*DT*: doubling time
